# Supplementary material for: A 24-month National Cohort Study examining long-term effects of COVID-19 in children and young people
Source: Commun Med (Lond). 2024 Dec 4;4:255. doi: 10.1038/s43856-024-00657-x (PMC11618575; doi:10.1038/s43856-024-00657-x)
Supplement: Supplementary file 2 — Supplementary Information [file 43856_2024_657_MOESM2_ESM.pdf]

Long-Term Effects of COVID-19 in Children and Young People: A 24-Month National Cohort Study

Terence Stephenson, Snehal M Pinto Pereira, Manjula D Nugawela, Emma Dalrymple, Anthony Harnden, Elizabeth Whittaker, Isobel Heyman, Tamsin Ford, Terry Segal, Trudie Chalder, Shamez N Ladhani, Kelsey McOwat, Ruth Simmons, Laila Xu, Lana Fox-Smith, CLoCk Consortium and Roz Shafran.

8 **Supplementary Table 1:** Characteristics (N(%)) at baseline of CYP by SARS-CoV-2 and vaccination status[a] at 24 months

	Initial-negatives (NN) (N=4,531)				Negative & infected (NP) (N=2,402)				Initial-positives (PN) (N= 5,177)				Positive & reinfected (PP) (N= 519)			
	Vaccine dose				Vaccine dose				Vaccine dose				Vaccine dose			
	0 n (%)	1 n (%)	2 n (%)	3+ n (%)	0 n (%)	1 n (%)	2 n (%)	3+ n (%)	0 n (%)	1 n (%)	2 n (%)	3+ n (%)	0 n (%)	1 n (%)	2 n (%)	3+ n (%)
**Prevalence**	452 (10.0)	371 (8.2)	2,115 (46.7)	1,593 (35.1)	348 (14.5)	232 (9.7)	1,213 (50.5)	609 (25.3)	597 (11.5)	386 (7.5)	2,593 (50.1)	1,601 (30.9)	113 (21.8)	58 (11.2)	237 (45.7)	111 (21.4)
**Sex**																
Male	192 (12.3)	138 (8.9)	783 (50.3)	445 (28.5)	120 (14.6)	88 (10.7)	452 (55.00	162 (19.7)	235 (12.8)	149 (8.1)	991 (53.9)	465 (25.3)	49 (27.2)	16 (8.9)	86 (47.8)	29 (16.1)
Female	260 (8.7)	233 (7.8)	1,332 (44.8)	1,148 (38.6)	228 (14.4)	144 (9.1)	761 (48.2)	447 (28.3)	362 (10.8)	237 (7.1)	1,602 (48.0)	1,136 (34.0)	64 (18.9)	42 (12.4)	151 (44.5)	82 (24.2)
**Age at index test (years)**																
11-14	289 (15.3)	196 (10.4)	1,232 (65.2)	173 (9.2)	233 (18.4)	151 (11.9)	776 (61.3)	106 (8.4)	360 (15.4)	195 (8.3)	1,577 (67.3)	210 (8.9)	82 (31.40	30 (11.5)	136 (52.1)	13 (5.0)
15-17	163 (6.2)	175(6.6)	883 (33.4)	1,420 (35.2)	115 (10.1)	81 (7.1)	437 (38.5)	503 (44.3)	237 (8.4)	191 (6.7)	1,016 (35.8)	1,391 (49.1)	31 (12.0)	28 (10.8)	101 (39.2)	98 (38.0)
**Ethnicity**																
White	280 (8.4)	228 (6.8)	1,547 (46.4)	1,277 (38.3)	258 (13.3)	183 (9.4)	982 (50.4)	524 (26.9)	363 (9.4)	249 (6.5)	1,960 (50.8)	1,284 (33.3)	80 (20.1)	43 (10.8)	182 (45.6)	94 (23.6)
Asian or Asian British	98 (13.6)	83 (11.6)	351 (48.9)	186 (25.9)	32 (13.5)	24 (10.1)	130 (54.6)	52 (21.9)	112 (14.2)	77 (9.8)	396 (50.2)	204 (25.8)	22 (28.9)	7 (9.2)	35 (46.1)	12 (15.8)
Mixed	25 (10.4)	20 (8.3)	113 (46.9)	83 (34.4)	27 (21.1)	14 (10.9)	65 (50.8)	22 (17.2)	45 (17.1)	22 (8.4)	122 (46.4)	74 (28.1)	7 (25.0)	4 (14.3)	12 (42.9)	5 (17.9)
Black, African or Caribbean	32 (20.3)	31 (19.6)	75 (47.5)	20 (12.6)	23 (40.4)	8 (14.0)	21 (36.8)	5 (8.8)	45 (29.8)	23 (15.2)	61 (40.4)	22 (14.6)	1 (11.1)	3 (33.3)	5 (55.6)	0 (0.0)
Other	9 (16.9)	4 (7.5)	20 (37.7)	20 (37.7)	7 (25.9)	2 (7.4)	13 (48.2)	5 (18.5)	21 (24.1)	12 (13.8)	38 (43.7)	16 (18.4)	1 (50.0)	1 (50.00	0 (0.0)	0 (0.0)
Prefer not to say	8 (27.6)	5 (17.2)	9 (31.0)	7 (24.1)	1 (20.0)	1 (20.0)	2 (40.0)	1 (20.0)	11 (35.5)	3 (9.7)	16 (51.6)	1 (3.23)	2 (40.0)	0 (0.00	3 (60.0)	0 (0.0)
**Region**																
East Midlands	19 (6.0)	21 (6.7)	143 (45.4)	132 (41.9)	29 (16.4)	18 (10.2)	80 (45.2)	50 (28.3)	45 (11.4)	23 (5.8)	190 (47.9)	138 (34.8)	7 (16.7)	7 (16.7)	19 (45.2)	9 (21.4)
East of England	84 (8.5)	84 (8.5)	459 (46.4)	362 (36.6)	78 (12.8)	55 (9.0)	339 (55.7)	137 (22.5)	104 (11.8)	61 (6.9)	442 (50.3)	271 (30.8)	12 (16.4)	7 (9.6)	42 (57.5)	12 (16.4)
**Region**																

London	97 (10.1)	92 (9.6)	482 (50.2)	289 (30.1)	81 (19.4)	42 (10.1)	204 (48.9)	90 (21.6)	143 (14.5)	88 (8.9)	484 (49.0)	273 (27.6)	26 (27.9)	12 (12.9)	40 (43.0)	15 (16.1)
North East	13 (8.1)	15 (9.4)	76 (47.5)	56 (35.0)	10 (14.9)	7 (10.5)	30 (44.8)	20 (29.9)	17 (8.1)	16 (7.6)	112 (53.3)	65 (30.9)	2 (11.1)	4 (22.2)	6 (33.3)	6 (33.3)
North West	78 (15.8)	39 (7.9)	216 (43.7)	161 (32.6)	45 (20.5)	22 (10.0)	94 (42.7)	59 (26.8)	74 (12.3)	52 (8.7)	300 (50.0)	174 (29.0)	13 (19.4)	4 (6.0)	33 (49.2)	17 (25.4)
South East	58 (7.9)	36 (4.9)	329 (44.8)	311 (42.3)	41 (9.6)	34 (8.0)	233 (54.6)	119 (27.9)	80 (9.1)	49 (5.6)	433 (49.0)	321 (36.4)	16 (19.3)	12 (14.5)	35 (42.2)	20 (24.1)
South West	13 (6.7)	12 (6.2)	93 (48.4)	74 (38.5)	15 (11.8)	11 (8.7)	55 (43.3)	46 (36.2)	22 (7.8)	20 (7.0)	145 (51.10	97 (34.2)	9 (20.5)	2 (4.6)	18 (40.9)	15 (34.1)
West Midlands	58 (7.9)	43 (10.7)	183 (45.4)	119 (29.5)	28 (14.1)	25 (12.6)	98 (49.3)	48 (24.1)	67 (12.9)	44 (8.5)	265 (51.1)	143 (27.6)	15 (30.0)	7 (14.0)	22 (44.0)	6 (12.0)
Yorkshire and the Humber	32 (11.3)	29 (10.2)	134 (47.2)	89 (31.3)	21 (13.2)	18 (11.3)	80 (50.3)	40 (25.2)	45 (10.7)	33 (7.9)	222 (53.0)	119 (28.4)	13 (26.5)	3 (6.1)	22 (44.9)	11 (22.4)
**IMD quintile**																
1 (most deprived)	142 (18.8)	110 (14.5)	337 (44.6)	167 (22.1)	94 (28.1)	45 (13.4)	131 (39.1)	65 (19.4)	172 (20.8)	98 (11.9)	387 (46.9)	168 (20.4)	33 (30.6)	19 (17.6)	39 (36.1)	17 (15.7)
2	102 (12.5)	82 (10.1)	394 (48.3)	237 (29.1)	72 (18.8)	48 (12.5)	201 (52.5)	62 (16.2)	136 (14.7)	109 (11.7)	455 (49.0)	228 (24.6)	25 (27.5)	13 (14.3)	37 (40.7)	16 (17.6)
3	73 (8.5)	71 (8.3)	413 (48.1)	302 (35.2)	72 (15.6)	42 (9.1)	238 (51.7)	108 (23.5)	112 (11.9)	71 (7.8)	475 (50.5)	283 (30.1)	29 (29.6)	7 (7.1)	42 (42.9)	20 (20.4)
4	78 (8.1)	45 (4.7)	454 (47.5)	387 (40.2)	51 (9.7)	49 (9.3)	282 (53.6)	144 (27.4)	79 (7.1)	64 (5.8)	570 (51.4)	395 (35.6)	14 (13.3)	15 (14.3)	51 (48.6)	25 (23.8)
5 (least deprived)	57 (5.0)	63 (5.5)	517 (45.5)	500 (43.9)	59 (8.5)	48 (6.9)	361 (51.7)	230 (32.9)	98 (7.1)	44 (3.2)	706 (51.3)	527 (38.3)	12 (10.3)	4 (3.4)	68 (58.1)	33 (28.2)

[a]Those who did not respond to the vaccine question (n=3) were excluded.

Supplementary Table 2: Characteristics of CYP 24 months post index-test by SARS-CoV-2 and vaccination status at 24 months (N(%) or Mean(SD))

	Initial test-negatives (NN) N=4,531 Vaccine dose				Negative & infected (NP) N=2,402 Vaccine dose				Initial test-positives (PN) N=5,177 Vaccine dose				Positive-reinfected (PP) N=519 Vaccine dose			
	0 (n=452)	1 (n=371)	2 (n=2,115)	3+ (n=1,593)	0 (n=348)	1 (n=232)	2 (n=1,213)	3+ (n=609)	0 (n=597)	1 (n=386)	2 (n=2,593)	3+ (n=1,601)	0 (n=113)	1 (n=58)	2 (n=237)	3+ (n=111)
**Number of symptoms**																
0	213 (47.1)	145 (39.1)	1,017 (48.1)	717 (45.0)	126 (36.2)	90 (38.8)	514 (42.4)	237 (38.9)	221 (37.0)	133 (34.5)	1,017 (39.2)	598 (37.4)	48 (42.5)	21 (36.2)	84 (35.4)	31 (27.9)
1	66 914.6)	68 (18.3)	325 (15.4)	263 (16.5)	54 (15.5)	45 (19.4)	208 (17.1)	99 (16.3)	85 (14.2)	54 (14.0)	467 (18.0)	280 (17.5)	25 (22.1)	6 (10.3)	44 (18.6)	21 (18.9)
2	53 (11.7)	36 (9.7)	225 (10.6)	167 (10.5)	49 (14.1)	28 (12.1)	130 (10.7)	65 (10.7)	76 (12.7)	48 (12.4)	310 (12.0)	217 (13.6)	9 (8.0)	7 (12.1)	21 (8.9)	13 (11.7)
3	32 (7.1)	29 (7.8)	152 (7.2)	119 (7.5)	27 (7.8)	13 (5.6)	93 (7.7)	50 (8.2)	49 (8.2)	44 (11.4)	226 (8.7)	137 (8.6)	6 (5.3)	5 (8.6)	18 (7.6)	12 (10.8)
4	25 (5.5)	23 (6.2)	113 (5.3)	100 (6.3)	16 (4.6)	12 (5.20	73 (6.0)	48 (7.9)	40 (6.7)	35 (9.1)	184 (7.1)	103 (6.4)	8 (7.1)	3 (5.2)	20 (8.4)	9 (8.1)
≥5	63 (13.9)	70 (18.9)	283 (13.4)	227 (14.3)	76 (21.8)	44 (19.0)	195 (16.1)	110 (18.1)	126 (21.1)	72 (18.60	389 (15.0)	266 (16.6)	17 (15.0)	16 (27.6)	50 (21.1)	25 (22.5)
**EQ5DY**																
Some/lots of mobility problems	37 (8.2)	24 (6.5)	115 (5.4)	136 (8.5)	32 (9.2)	19 (8.2)	53 (4.4)	41 (6.7)	43 (7.2)	29 (7.5)	129 (5.0)	91 (5.7)	8 (7.1)	2 (3.5)	20 (8.4)	7 (6.3)
Some/lots of self-care problems	21 (4.7)	13 (3.5)	73 (3.5)	91 (5.7)	22 (6.3)	17 (7.3)	41 (3.4)	30 (4.9)	21 (3.5)	21 (5.4)	73 (2.8)	53 (3.3)	7 (6.2)	4 (6.9)	13 (5.5)	0 (0)
Some/lots of problems with usual activities	70 (15.5)	47 (12.7)	228 (10.8)	231 (14.5)	73 (21.00	42 (18.1)	137 (11.3)	85 (14.0)	108 (18.1)	57 (14.8)	307 (11.8)	195 (12.2)	19 (16.8)	15 (25.9)	35 (14.8)	16 (14.4)
Some/lots of pain discomfort	87 (19.3)	73 (19.7)	354 (16.7)	336 (21.1)	84 (24.1)	61 (26.3)	216 (17.8)	124 (20.4)	147 (24.60	85 (22.0)	444 (17.1)	288 (18.0)	26 (23.0)	16 (27.6)	53 (22.4)	27 (24.3)
Very worried, sad/unhappy	36 (8.0)	29 (7.8)	144 (6.8)	153 (9.6)	28 (8.1)	25 (10.8)	89 (7.3)	61 (10.0)	39 (6.5)	34 (8.8)	187 (7.2)	114 (7.1)	10 (8.8)	8 (13.8)	19 (8.0)	9 (8.1)
**SDQ**																

Total Difficulties	12.2 (6.6)	12.6 (6.6)	11.9 (6.5)	12.9 (6.3)	12.9 (6.9)	13.1 (6.90	11.9 (6.5)	12.4 (6.2)	12.3 (6.5)	13.0 (6.8)	11.8 (6.4)	12.1 (6.3)	12.5 (7.4)	14.4 (7.2)	12.2 (6.9)	12.3 (6.3)
Emotional symptoms	3.7 (2.7)	4.0 (2.8)	3.7 (2.6)	4.4 (2.6)	4.1 (2.7)	4.0 (2.7)	3.8 (2.6)	4.2 (2.7)	3.8 (2.6)	4.0 (2.7)	3.7 (2.6)	4.1 (2.6)	3.7 (2.8)	4.7 (3.0)	4.1 (2.8)	4.4 (2.4)
Conduct problems	1.9 (1.6)	1.8 (1.5)	1.7 (1.6)	1.5 (1.5)	2.0 (1.7)	2.0 (1.8)	1.7 (1.6)	1.4 (1.4)	1.9 (1.7)	1.9 (1.7)	1.6 (1.6)	1.4 (1.5)	2.0 (1.8)	2.3 (1.8)	1.6 (1.7)	1.6 (1.6)
Hyperactivity /inattention	4.2 (2.5)	4.3 (2.6)	4.2 (2.6)	4.4 (2.6)	4.4 (2.8)	4.7(2.7)	4.2 (2.6)	4.4 (2.5)	4.2 (2.6)	4.4 (2.6)	4.2 (2.6)	4.2 (2.6)	4.3 (2.7)	4.6 (2.6)	4.2 (2.7)	4.2 (2.7)
Peer relationship problem	2.5 (1.9)	2.5 (1.9)	2.3 (1.9)	2.6 (1.9)	2.5 (1.9)	2.4 (1.9)	2.1 (1.9)	2.3 91.9)	2.4 (1.8)	2.6 (1.9)	2.2 (1.8)	2.4 (1.9)	2.4 (2.0)	2.8 (2.4)	2.3 (2.0)	2.2 (1.7)
**SWEMBS**	20.9 (4.3)	21.0 (4.3)	21.4 (4.2)	20.8 (3.9)	20.5 (3.9)	21.1 (4.2)	21.3 (4.2)	21.1 (4.1)	21.1 (4.3)	20.7 (4.3)	21.4 (4.0)	21.2 (3.8)	21.6 (5.5)	20.3 (3.7)	21.2 (4.1)	21.0 (3.8)
**Chalder fatigue scale**	13.3 (5.7)	14.4 (5.6)	13.8 (5.1)	14.5 (5.2)	14.5 (6.2)	14.4 (5.5)	13.9 (5.0)	14.9 (5.5)	14.5 (5.9)	14.2 (5.7)	14.0 (5.1)	14.6 (5.1)	14.4 (6.2)	16.3 (6.40	14.2 (5.4)	15.9 (5.7)
**Self-rated health[a]**	85 (73, 95)	80 (70, 95)	85 (75, 95)	80 (70, 90)	85 (70, 95)	85 (70, 90)	85 (70, 95)	80 (70, 90)	85 (70, 95)	80 (70, 90)	85 (75, 95)	80 (70, 90)	85 (70, 95)	83 (70, 90)	85 (70, 90)	80 (70, 90)
**Symptom severity[a,b]**	50 (30, 70)	50 (30, 70)	50 (30, 70)	50 (30, 60)	60 (40, 70)	50 (30, 60)	50 (30, 60)	50 (30, 60)	50 (30, 70)	50 (30, 70)	50 (30, 60)	50 (30, 70)	50 (30, 70)	60 (35, 60)	45 (20, 60)	55 (40, 70)
**Symptom impact[a,c]**	50 (20, 70)	40 (20, 70)	40 (10, 70)	40 (20, 70)	50 (30, 70)	50 (30 , 70)	40 (20, 70)	50 (20, 70)	50 (20, 70)	45 (20, 70)	40 (20, 70)	40 (10, 70)	50 (20, 80)	40 (20, 60)	40 (10, 70)	40 (20, 60)

Note: Those who did not respond to the vaccine question (n=3) were excluded.
Note. SDQ = Strengths and Difficulties Questionnaire; SWEMBS = Short Warwick-Edinburgh Mental Wellbeing Scale.
A higher SDQ score indicates more problems; a higher SWEMWBS score indicates better mental well-being; a higher fatigue score is more severe.
[a]Reported as median(IQR), scored on a scale of 0 (worst) to 100 (best) for self-rated health; 0 (not severe at all) to 100 (extremely severe) for symptom severity; 0 (no impact) to 100 (extreme impact) for symptom impact; [b]based on sub-sample N=4,977; [c]based on sub-sample N=4,972.

Supplemental Figure 1: Timeline of enrolment into the CLoCk study for the analytical sample

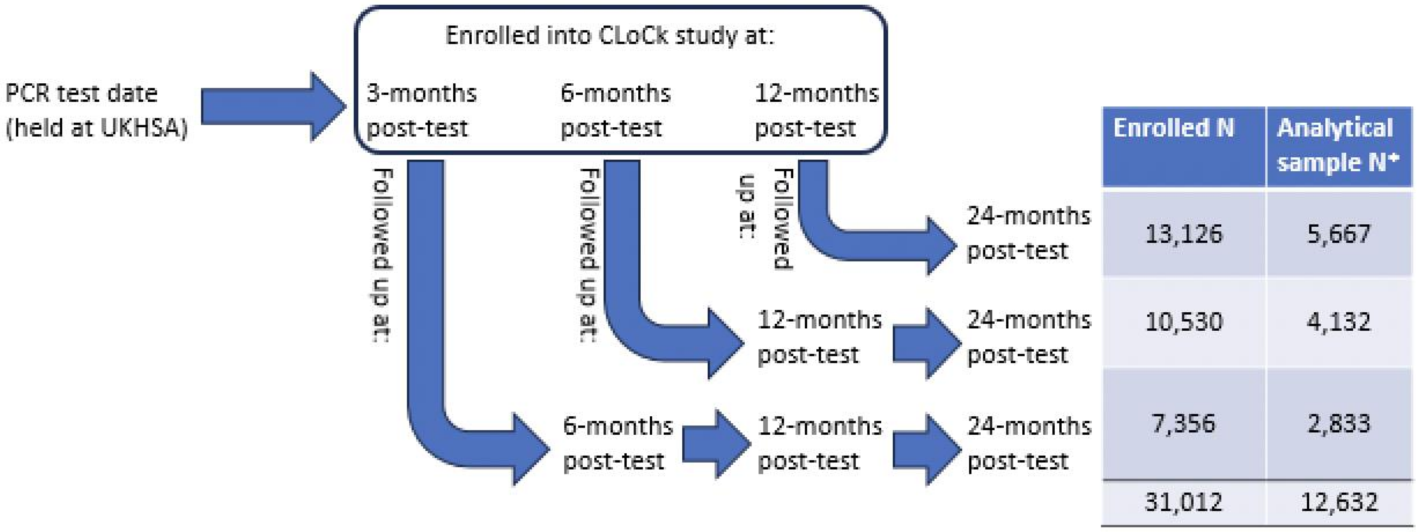

*The analytical sample includes children and young people who enrolled (at 3-, 6- or 12-months post-PCR-testing) AND filled int the 24-month questionnaire.
